# Hematological indices derived from complete blood count and unfavorable outcomes in patients under-going peritoneal dialysis

**DOI:** 10.1590/2175-8239-JBN-2025-0017en

**Published:** 2025-09-12

**Authors:** Taluane Vívian Gomes Alves, Luciane Teixeira Passos Giarola, Wander Valadares de Oliveira, Danyelle Romana Alves Rios

**Affiliations:** 1 Universidade Federal de São João del-Rei, Divinópolis, MG, Brazil.; 2 Universidade Federal de São João del-Rei, Departamento de Matemática e Estatística, São João del Rei, MG, Brazil.; 3 Universidade do Estado de Minas Gerais, Passos, MG, Brazil.

**Keywords:** Biomarkers, Peritoneal Dialysis, Mortality, Cox Models

## Abstract

**Introduction:**

Understanding the inflammatory processes that are associated with the risk of mortality in patients undergoing peritoneal dialysis (PD) may help guide clinical decision-making and risk and mortality stratification in this population.

**Objective:**

To evaluate the association of hematological indices derived from complete blood count with unfavorable outcomes in patients undergoing PD.

**Methods:**

Prospective cohort with 43 patients undergoing PD follow up for 18 months. Complete blood count data were collected from medical records and the hematological indices were calculated for all participants in the four follow-up waves. Associations between these indices and classic inflammatory markers were investigated by correlation analyses. Patient survival was estimated by the Kaplan Meier method (K-M) after dividing the patients into two groups based on the median as the cut-off point for each hematological index. The Cox model with competitive-risk framework was used to evaluate the influence of indices on survival.

**Results:**

The AISI and SIRI indices had a significant positive correlation with global leukocytes (r = 0.74 and r = 0.71, respectively, p < 0.001). Only AISI and SII showed K-M significant estimates indicating greater survival for AISI ≤149.61 and SII ≤722.80. In the Cox regression model, patients who presented AISI above 149.6 and SII above 722.80 had 9.38 and 4.0 times, respectively, higher risk of death or transfer to HD than other patients.

**Conclusion:**

AISI and SII were independently associated with the risk of unfavorable outcomes in PD patients.

## Introduction

Chronic kidney disease (CKD) is an important public health problem because of its high prevalence, economic impact, and high morbidity and mortality^1^. Although CKD treatments have improved the condition over time, the chronic inflammation and oxidative stress in patients accelerate the process of morbidity and mortality^2^.

Peritoneal dialysis (PD) is one of the renal replacement therapy (RRT) options in which the peritoneum, the membrane that covers the main organs of the abdomen, is used for blood filtration. The proper functioning of PD depends on the structural and functional integrity of the peritoneal membrane. Peritonitis (peritoneum inflammation) related to PD remains the main complication and challenge for the long-term success of PD and the main cause of PD failure and transfer to hemodialysis (HD)^3,4^.

In the routine monitoring of PD patients, cheaper, more convenient, and more effective measurements are needed for stratification of the risk of unfavorable outcomes^5^. Thus, new markers have been characterized as potential prognostic factors for cardiovascular disease (CVD) mortality and all-cause mortality in patients undergoing PD and HD, and also for other clinical conditions^5,6,7,8^. Examples of such markers are hematological indices from complete blood count, which can be obtained by simple calculations based on neutrophil, lymphocyte, platelet, and monocyte blood count^9^. Hematological indices include: neutrophil-lymphocyte ratio (NLR), platelet-lymphocyte ratio (PLR), derivative neutrophil-lymphocyte ratio (dNLR), monocyte-lymphocyte ratio (MLR), aggregate index of systemic inflammation (AISI), systemic inflammatory index (SII), and the systemic inflammatory response index (SIRI)^9,10^.

Inflammation is a common feature of CKD, often associated with elevated levels of inflammatory markers such as the pro-inflammatory cytokines IL-6, IL-1, and tumor necrosis factor-α (TNF-α), as well as anti-inflammatory cytokines such as IL-10. In a study evaluating the potential of several inflammatory biomarkers in the diagnosis and staging of CKD, circulating levels of IL-6 and TNFR2 were significantly elevated in all stages of the disease^11^. However, these markers are not routinely assessed due to the high costs involved^5^.

Serum ferritin, also considered an inflammatory marker, may be increased in inflammatory conditions such as CKD, independently of iron stores, and is positively correlated with the severity of inflammation^12^. Global leukocyte, another widely used inflammatory marker, is employed in different clinical contexts^13,14^. As all proportions of these indices are derived from the global leukocyte, there is a mathematical relationship between them. Ferritin and global leukocyte were significantly higher in patients who died compared to survivors in a study that evaluated hematologic indices (NLR, MLR, PLR, dNLR, AISI, SIRI, and SII) in predicting mortality in 1,792 elderly and non-elderly patients who tested positive for COVID-19^15^.

Understanding the inflammatory processes that are associated with the risk of mortality in patients undergoing PD is fundamental for the control of systemic inflammation^16^. To guide clinical decision making in order to enable better prognosis, more affordable therapeutic and diagnostic resources that can identify possible complications of the disease and the risk of mortality are necessary^5^. Thus, the present study aims to evaluate the association of hematological indices derived from complete blood count with unfavorable outcomes (by all-cause mortality and transfer to HD) in patients undergoing PD.

## Method

### Design and Study Population

This is a prospective cohort study with PD patients at a nephrology center of a municipality in the state of Minas Gerais. It is a regional reference center for the treatment of kidney diseases and one of the largest nephrology centers in the country. Participants were monitored from August 2011 to February 2013.

Eligible patients were invited to participate in the study after routine outpatient consultation, conducted once a month. Of a total of 74 patients undergoing PD, 43 were considered eligible. The inclusion criteria were: being in PD for at least 90 days, clinically stable (without episodes of peritonitis, hospitalization, or acute diseases one month before, during, or one month after evaluation), and 18 years old or older. Exclusion criteria were acute diseases, autoimmune diseases, neoplasms, being seropositive for HIV, pregnancy, and unable to sign the free and informed consent due to psychiatric disease or mental disorder.

**Figure 1:**
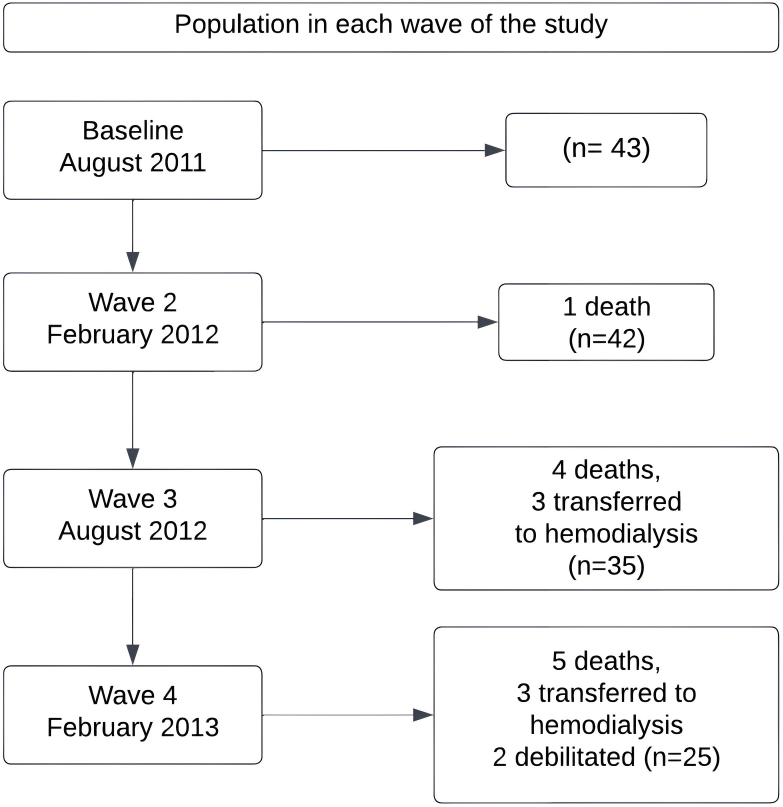
Population in each wave of the study.

The baseline evaluation, considered as wave 1 of the study (August 2011), included 43 patients. Wave 2 occurred in February 2012 and there was one loss due to death. In wave 3, which occurred in August 2012, there were seven losses (16.3%), four due to death (9.3%) and three due to transfer to HD (7%). Wave 4 occurred in February 2013 and during follow-up there were eight losses (20.9%) - five deaths (11.6%) and three transfers to HD (7.0%) and two patients were debilitated (2.3%), thus finalizing the study with 25 patients (Figure 1).

### Data Collection

Data collection was performed at the time of the routine consultation that occurred every six months. Information was collected from patients’ medical records on sociodemographic data, health conditions, routine laboratory tests, medicine use, peritonitis registration, complications that occurred during the period of the survey, total PD duration time and other dialytic therapies, and date of death. Data on mortality, time of death, and HD transfer time was collected through patients’ medical records, which were in the hospital archive. The information was checked against the Nefrodata Information System of the Hospital’s Nephrology Unit.

Complete blood count data were collected from the medical records. Hematological indices were calculated for all participants in the four follow-up waves, namely: NLR: neutrophils/lymphocytes ratio; dNLR: neutrophils/global leukocytes – neutrophils; PLR: platelets/lymphocytes; MLR: monocytes/lymphocytes; AISI: neutrophils × monocytes × platelets/lymphocytes; SII: platelets × neutrophils/lymphocytes and SIRI: neutrophils × monocytes/lymphocytes.

The following variables were also collected: sex, age, primary disease, total PD time, time in the survey, number of peritonitis episodes, HD before PD, HD or PD therapy time, transferred to HD, time to death (from the beginning of the study to death), body mass index (BMI), presence of diabetes mellitus and hypertension, systolic and diastolic blood pressure, fasting blood glucose, creatinine, serum iron, total iron binding capacity, iron saturation index, ferritin, total cholesterol and fractions, triglycerides, alanine aminotransferase, alkaline phosphatase, total protein, albumin, globulin, calcium, phosphorus, potassium, parathyroid hormone (PTH).

The study was approved by the Ethics and Research Committees of the Federal University of São João Del-Rei and the São João de Deus Hospital-CAAE-19284613.5.0000.5545.

### Statistical Analysis

Descriptive data were presented as number and frequency (%) for categorical data, average and standard deviation for normal continuous data, and median and interquartile intervals for asymmetrical data. The Shapiro-Wilk test was used to test the normality of the data. Pearson’s correlation test was used to evaluate the association between classic inflammatory markers (ferritin, global leukocytes, IL-6, and IL-10) and hematological indices.

Patient survival was estimated by the Kaplan Meier method. This method was also used to estimate the survival of patients when dichotomized by the median for each of the hematological indices: lower than or equal to the median and higher than the median. Comparison of the survival curves between the groups of each index was performed through the log-rank test.

To assess the influence of hematological indices on survival, the Cox proportional hazards model with competitive risks was used. Two outcomes were considered: death and transfer to HD treatment. The analysis procedure followed characterization analogous to the classic Cox regression model^17^. Thus, models were constructed considering one of the seven hematological indices in each model and the covariates sex, age, total time on PD, number of episodes of peritonitis, HD before PD, BMI, presence of diabetes mellitus, systolic blood pressure, diastolic blood pressure, fasting blood glucose, creatinine, and arterial hypertension.

A prior selection of the model variables was performed by the “backward” process, using the Akaike Information Criterion (AIC), considering the variables that make up the model with the lowest AIC value. The significance of these variables was assessed using the Wald test^18^. Those that did not present statistical significance were excluded from the model one at a time until a model was obtained in which all variables influenced the risk of the outcomes. From this step on, the previously excluded variables were reintegrated one at a time into the model obtained and their significance was again assessed using the Wald test and also using the likelihood ratio test when comparing models with and without the aforementioned variable. The variables that are known in clinical practice to be important in explaining death or transfer to HD were included in the final model even if they were not statistically significant. The estimates for the parameters, performed using the partial maximum likelihood method, refer to the common (mean) effect of the variables for the two outcomes.

Schoenfeld residual plots were constructed to assess the Cox model’s assumption of proportionality of risks using the Pearson’s correlation coefficient between residuals and time, and a hypothesis test for this correlation was performed. High significance probability values are desired, since they indicate proportional risks. Martingale and deviance residuals were used to assess the adequacy of the adjusted model.

The analyses were performed at a 5% significance level and conducted using R software (2023), with the aid of the ‘SURVIVAL’ and ‘MASS’ packages, and STATA software version 14.0^19,20^.

## Results

The baseline characteristics of the study population are presented in Table 1. The mean age of the patients was 63 years, 51.2% were male, and the median BMI was 24.6. Ten participants (23.3%) had already undergone HD therapy before switching to PD. The mean systolic blood pressure was 142.0 and the median diastolic blood pressure was 80.0. Among the known etiologies, the main cause of CKD was diabetes mellitus (30.3%).

**Table 1:** Sociodemographic, clinical and laboratory characteristics of the study population

Variables	Total	Reference values
Age (Years)	63.0 ± 15.3	
Sex		
Male [n (%)]	22 (51.2%)	
BMI (kg/m^2^)	24.6 (21.3; 26.3)	
Primary causes of CKD [n (%)]		
Diabetes	13 (30.3%)	
Systemic arterial hypertension	7 (16.3%)	
CGN	2 (4.6%)	
PKD, CAKUT	3 (7%)	
Unknown aetiologies	18 (41.8%)	
Systolic pressure (mmHg)	142.0 ± 21.4	
Diastolic pressure (mmHg)	80 (80; 80)	
Total patients with peritonitis recorded (n) in 18 months [n (%)]		
0 peritonitis	28 (65.1%)	
1 peritonitis	12 (57.1%)	
2 peritonitis	1 (4,8)	
3 peritonitis	1 (4.8%)	
4 peritonitis	1 (4.8%)	
Patients with peritonitis [n (%)]	15 (34.9%)	
Total time on PD (months)	46.4 (31.0; 62.7)	
HD before PD [n (%)]	10 (23.3%)	
Time on HD or PD therapy (months)	45.8 ± 20.3	
Time participating in the study (months)	16.4 ± 4.1	
Deaths [n (%)]	10 (23.3%)	
Time to death (months)	12.5 ± 4.2	
Transferred to HD [n (%)]	6 (13.9%)	
Has been on PD for over 36 months [n (%)]	17 (39.5%)	
Fasting blood glucose (mg/dL)	102 (82; 146)	Less than 99 mg/dL
Serum creatinine (mg/dL)	9.47 ± 4.26	M: 1 (0.7 to 1.2 mg/dL) W: 0.8 (0.5 to 1.0 mg/dL)
Serum iron (mcg/dL)	64.88 ± 26.3	M: 65 mcg/dL to 175 mcg/dL W: 50 mcg/dL to 170 mcg/dL
Total iron binding capacity (mcg/dL)	209 (189; 253)	M: 69 mcg/dL to 240 mcg/dL W: 70 mcg/dL to 310 mcg/dL
Transferrin saturation index (%)	27.6 ± 13.3	20.0 to 55.0%
Ferritin (ng/mL)	169 (100; 482)	M: 23 to 336 ng/mL W: 12 to 306 NG/mL
Total cholesterol (mg/dL)	188.9 ± 66.0	< 190 mg/dL
HDL-C (mg/dL)	40 (34; 48)	M: > 40 mg/dL W: > 50 mg/dL
LDL-C (mg/dL)	114.4 ± 53.9	<130 mg/dL
Triglycerides (mg/dL)	150 (110; 208)	<150 mg/dL
Alanine aminotransferase (U/L)	19 (17; 25)	M: < 45 U/L W: < 34 U/L
Alkaline phosphatase (U/L)	93 (79; 117)	40 U/L to 150 U/L
Total protein (g/dL)	6.1 ± 0.6	6.0 to 8.0 g/dL
Albumin (g/dL)	3.2 ± 0.4	3.5 g/dL to 5.2 g/dL
Globulin (g/dL)	2.9 ± 0.4	1.5 to 3.5 g/dL
Calcium (mg/dL)	9.3 ± 0.7	8.8 to 10.4 mg/dL
Phosphorus (mg/dL)	5 (4.1; 6.4)	M: 2.4 to 4.6 mg/dL W: 2.3 to 4.3 mg/dL
Potassium (mmol/L)	4.5 ± 0.8	3.5 to 5.0 mmol/L
Parathyroid hormone (pg/mL)	184 (68; 375)	12.0 pg/ml to 88.0 pg/ml
Hemoglobin (g/dL)	11.7 ± 1.9	M: 14.9 g/dL (13.0–16.9) W: 13.2 g/dL (11.5–14.9)
Global leukocyte (x 10^9^/L)	6.4 ± 2.5	4.0 to 10.0 x 10^9^/L
Neutrophils (x 10^9^/L)	4.4 ± 1.7	2.0 to 7.0 x 10^9^/L
Bands (x 10^9^/L)	0.2 (0;0)	0 to 1.2 x 10^9^/L
Monocytes (x 10^9^/L)	0.2 (0.2; 0.3)	0.2 to 1.2 x 10^9^/L
Basophils (x 10^9^/L)	0.6 (0;0)	0 to 0.22 x 10^9^/L
Lymphocytes (x 10^9^/L)	1.4 (1.2; 1.9)	1.0 a 3.0 x 10^9^/L
Platelets (x 10^9^/L)	219.9 ± 81.7	150 to 450 x 10^9^/L

Abbreviations – BMI: body mass index; CKD: chronic kidney disease; CGN: chronic glomerulonephritis; PKD: polycystic kidney disease; CAKUT: congenital abnormalities of the kidneys and urinary tract; PD: peritoneal dialysis; HD: hemodialysis; HDL-C: high-density lipoprotein-cholesterol; LDL-C: low-density lipoprotein-cholesterol; W: women; M: men.

Notes – The results are presented as mean and standard deviation for data with normal distribution, according to the Shapiro-Wilk test, and median (quartiles) for data with asymmetric distribution. Categorical variables are presented as proportions: n (%).

The mean duration of PD was 53.5 months, with 50% of patients having a duration of up to 46.4 months. Twenty-one episodes of peritonitis were recorded, with 15 (34.9%) patients having peritonitis during the monitoring period. The mean duration of patient participation in the study was 16.4 months. At the end of the monitoring period, there were 10 (23.3%) deaths and 6 (13.9%) patients had been transferred to HD.

Table 2 presents the results obtained for Pearson’s correlation coefficients (r) between classical inflammatory markers (ferritin, total leukocyte count, IL-6, and IL-10) and hematological indices at baseline. The AISI and SIRI indices had a high, positive, and statistically significant correlation with total leukocyte count (r = 0.74 and r = 0.71, respectively). The PLR index showed a low, negative, and significant correlation with total leukocyte count (r = -0.32). The SII index showed a low, positive, and significant correlation with total leukocyte count (r = 0.45). The other indices did not show statistically significant correlations. The correlation scale used is for the values of the module correlation, being classified as very low for values equal to or less than 0.25, low for values from 0.26 to 0.49, moderate between 0.50 and 0.69, high between 0.70 and 0.89, and very high between 0.90 and 1.00^21^.

**Table 2:** Correlation analysis between the markers ferritin, global leukocyte count, il-6 and il-10 and the haematological indices at the baseline of the study

Markers	Ferritin	Global leukocytes	IL-6	IL-10
NLR	–0.14	0.22	–0.14	–0.09
dNLR	–0.17	0.19	–0.10	0.01
PLR	–0.11	–0.32*	–0.07	0.10
MLR	–0.15	0.16	–0.18	0.07
AISI	–0.19	0.74*	–0.11	–0.07
SII	–0.17	0.45*	–0.15	–0.03
SIRI	–0.20	0.71*	–0.17	–0.10

Abbreviations – NLR: neutrophil lymphocyte ratio; dNLR: derived neutrophil lymphocyte ratio; PLR: platelet lymphocyte ratio; MLR: monocyte lymphocyte ratio; AISI: Aggregate Systemic Inflammation Index; SII: Systemic Inflammatory Index; SIRI: Systemic Inflammatory Response Index; IL-6: interleukin 6; IL-10: interleukin 10.

Note – *p < 0.001.

**Figure 2:**
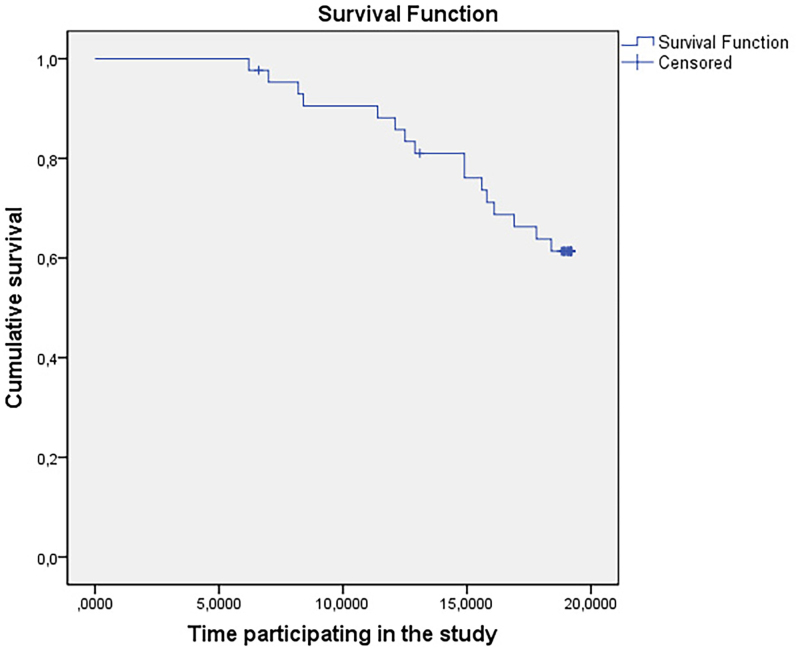
Kaplan-Meier survival estimate curve.

Figure 2 presents the survival estimates of the 43 patients obtained by Kaplan Meier estimator. In this figure it can be observed that the estimates for survival had values above 0.6. This can be explained by the short period of patient monitoring (about 18 months).

**Figure 3:**
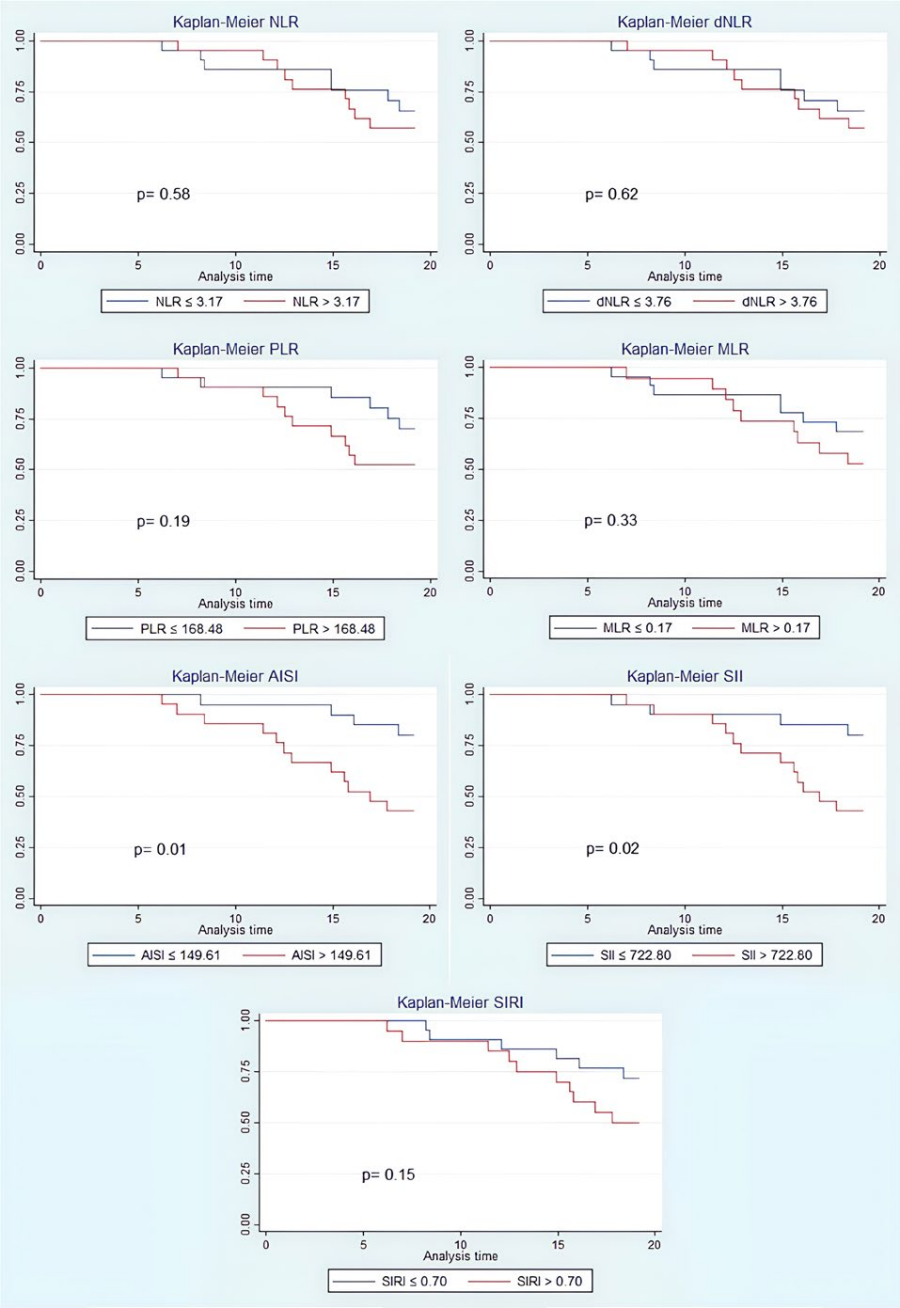
Kaplan-Meier survival estimate curves for all indices categorized according to median values.

Figure 3 shows survival estimates from the Kaplan Meier estimator for each of the groups obtained according to the median values at the baseline of each of the indices. The medians for each index were: NLR = 3.17; dNLR = 3.76; PLR = 168.48; MLR = 0.15; AISI = 149.61; SII = 722.80; and SIRI = 0.70. For the AISI (p = 0.01) and SII (p = 0.02) indices, the survival was higher in patients below the median value, being statistically significant. NLR, dNLR, PLR, MLR, and SIRI were not statistically significant (p = 0.58, p = 0.62, p = 0.19, and p = 0.33, p = 0.15, respectively).

Considering the COX model for competitive risks, it was possible to obtain adjustment for the AISI and SII indices through two models. The other indices did not have statistical significance for the risk of occurrence of the outcomes. The adjusted models included the variables of interest, regardless of their significance.

Estimates of the final model adjusted for AISI are presented in Table 3, together with their respective hazard ratio (HR) and results obtained for the Wald test. Total PD (p = 0.03), age (p = 0.01), creatinine (p < 0.01), and the number of episodes of peritonitis (p = 0.02) were statistically significant. Total PD time and age had a hazard ratio close to 1 (HR = 0.97 and HR = 1.09, respectively). Each month the patient remained in PD reduced the risk of death or transfer to hemodialysis by 3%. Each year of life, risk of death, or transfer to HD increased by 9%. Each 1 unit increase in creatinine increased the risk of death or transfer to HD by 38% (HR = 1.38; p = 0.01). With each episode of diagnosed peritonitis, the risk of death or transfer to HD increased by 2.34 times. Patients with AISI above 149.6 had 9.38 times higher risk of death or transfer to HD than patients with AISI up to 149.6. Sex and HD variables before PD were not statistically significant.

**Table 3:** Estimates obtained for adjusted model parameters, considering the competitive risks of death and transfer to hemodialysis and the aisi index

Parameter	Estimate	Deviation	Wald Statistic	p value	HR	CI_95%_(HR)
Total time in PD	–0.03	0.01	–2.20	0.03*	0.97	(0.953; 0.997)
Sex	0.01	0.54	0.01	0.99	1.01	(0.352; 2.880)
Age	0.08	0.03	2.56	0.01*	1.09	(1.020; 1.161)
HD before PD	–1.09	0.99	–1.11	0.27	0.34	(0.049; 2.324)
Number of peritonitis episodes	0.85	0.37	2.28	0.02*	2.34	(1.127; 4.842)
Creatinine	0.32	0.11	3.01	< 0.01*	1.38	(1.119; 1.702)
AISI	2.24	0.78	2.86	< 0.01*	9.38	(2.025; 43.420)

Abbreviations – PD: peritoneal dialysis; HD: Hemodialysis; AISI: aggregate index of systemic inflammation.

Note – *Significant at 5%.

For the assessment of the proportional risk assumption, Figure S1 presents the graphs for Schoenfeld residuals and Table S1 shows the hypothesis test results for the Pearson correlation coefficient between Schoenfeld residuals and time for each variable of the model. No violations of proportional hazard (p values greater than 0.05) were observed. Although age had a lower value (0.07), there was no evidence that the hazard proportionality on all covariates was violated, given the overall value (0.67) for this assessment.

The Martingale and deviance residuals’ charts built to evaluate the overall quality of the adjusted model indicate the adequacy of the model, as the residuals are randomly distributed around zero (Figure S2), with the Martingale residuals at less than 1 and the deviance residuals at (–2, 2).

Estimates of the final model adjusted for SII, their respective hazard ratio, and results obtained by the Wald test are presented in Table 4. With each diagnosed peritonitis episode, the risk of death or transfer to HD increased by 80% (p = 0.04). Patients who had SII values above 722.8 had a risk of death or transfer to HD four times higher than other patients (p = 0.03). Total time in PD, sex, age, and HD before PD did not have statistically significant values.

**Table 4:** Estimates obtained for adjusted model parameters, considering the competitive risks of death and transfer to hemodialysis and the sii index

Parameter	Estimate	Deviation standard error	Wald statistic	p value	HR	IC_95%_(HR)
Total time in PD	–0.01	0.01	–1.18	0.24	0.99	(0.971; 1.007)
Sex	–0.17	0.53	–0.32	0.75	0.84	(0.297; 2.394)
Age	0.03	0.02	1.26	0.21	1.03	(0.985; 1.072)
HD before DP	–0.25	0.72	–0.34	0.73	0.78	(0.190; 3.217)
Number of peritonitis episodes	0.59	0.29	2.01	0.04*	1.80	(1.015; 3.182)
SII	1.39	0.65	2.14	0.03*	4.01	(1.127; 14.276)

Abbreviations – PD: peritoneal dialysis; HD: Hemodialysis; SII: Systemic inflammatory index.

Table S2 presents the hypothesis test results for Pearson’s correlation coefficient between Schoenfeld residuals and time, and Figure S3 presents the graphs for these residuals over time. The SII, while presenting a low value (0.08), can indicate proportional risks, as this p-value is greater than 5% and the chart corroborates this statement. The other variables also do not have evidence of violating the proportional hazards assumption. Also, the global assessment of the proportionality confirmed that this assumption was not violated (p-value of 0.38).

The Martingale and deviance residual charts built to evaluate the overall fit of the adjusted model indicate adequacy, as the residuals are randomly distributed around zero (Figure S4), with the Martingale residuals at less than 1 and the deviance residuals ranging between -3 and 3.

For the other indices, it was not possible to fit a Cox regression model for the risk of death or transfer to HD.

## Discussion

The present study identified that in an average monitoring period of 16.4 months, there were 10 deaths (23.3%) and six (13.9%) transfers to HD, indicating considerable impairment in the survival rate of PD patients, given the average follow-up time. The mortality rate was similar to the study of Liu et al.^6^, in which 25.5% of the 939 PD patients died in a median monitoring period of 27.5 months. Lu et al.^22^, however, found that 43% of the 86 PD patients died during the study period of an average duration of 25.6 months.

Survival estimates were also obtained for each of the hematological indices. PD patients with AISI ≤ 149.61 and SII ≤ 722.80 had a higher survival rate compared to patients with values above the average value (p = 0.01; p = 0.02, respectively). Based on COX models, AISI (95%CI (HR): 2.024; 43.420) and SII (95%CI (HR): 1.127; 14.276) were independently associated with increased risk of death or transfer to HD. The large confidence intervals of both indices can be explained by the study’s small sample size.

To date, no study had investigated the association between AISI and mortality in CKD. AISI ≥ 621.1 was shown to be a survival predictor in 85 COVID-19 hospitalized patients with CKD in stages 3-5 with an average age of 72.1 years (HR: 1.001; 95%CI: 1-1.001)^23^. AISI is based on the counts of lymphocytes, neutrophils, platelets, and monocytes. The distinct roles that these cells play in the immune response contribute to their ability to predict mortality risk in patients with COVID-19 and other pathologies^23^.

Lymphocytes act in the adaptive immune response by recognizing and destroying antigens in the body. The reduction of circulating lymphocytes is associated with recent infections, use of specific drugs, and health conditions related to the immune system. Lymphocytopenia leads to a persistent inflammatory state, because in addition to the immune system, lymphocytes are also responsible for regulating inflammation, acting bidirectionally with macrophages, mutually stimulating and amplifying the inflammatory response^24^.

The increase in circulating neutrophils suggests an acute or chronic inflammatory response. Increased neutrophils during systemic inflammation due to infections also drives the production of inflammatory mediators such as IL-1A, which stimulate megakaryocyte platelet production by increasing circulating platelet levels and contributing to inflammatory and immunological responses^25^. Neutrophils can cause inflammation in the renal parenchyma and inhibit lymphocyte activity by cessation of the regulatory response of T cells and activation of macrophages^26^.

Monocytes are cells produced in bone marrow that are highly reactive in inflammatory processes. By migrating to tissues, they become macrophages, acting on the immune response and, in response to a stimulus, they can intensify or reduce inflammation^23,26^. In addition, it has been described that the presence of monocyte fractions in steady state consistently increase during inflammation^27^.

There is still little evidence on the role of SII in CKD, specifically in PD patients, making it impossible to compare the results found. SII ≥ 1,063.39 proved to be an independent risk factor for mortality by all causes (HR: 1.48; 95%CI: (1.35–1.62), cardiovascular causes (HR: 1.64; 95%CI: 1.44–1.85), and cancer causes (HR: 1.50; 95%CI: 1.09–2.08) in 19,327 Chinese patients with CKD not on dialysis treatment (estimated GFR: <60 ml/min/1.73m^2^), monitored for approximately 4.5 years^28^. A study conducted in Brazil with patients with CKD stages 1 to 4 in the pre-dialysis period up to 18 years of age, evaluated the capacity of NLR, dNLR, LMR (lymphocyte-monocyte ratio), SIRI, AISI, and SII rates in predicting disease severity. Only LMR and SIRI presented promising results in the evaluation of inflammation; for SII there was no difference in comparing groups with CKD with each other or in relation to controls^26^. SII has previously been identified as to be a predictor of acute peritonitis treatment in a study of 138 Chinese patients with CKD (OR: 0.999; 95%CI: 0.998 –1.000), suggesting a different role from what was expected, acting as a protective factor in the prognosis of patients with acute peritonitis^29^.

With CKD progression and renal function degradation, the inflammatory process in the renal parenchyma increases. Moreover, other diseases and health conditions commonly present in these individuals can exacerbate the level of inflammation in the body^26,30^. Thus, hematological indices obtained by a higher combination of cells such as AISI (neutrophils, monocytes, platelets, and lymphocytes) and SII (platelets, neutrophils, and lymphocytes) may be more useful in evaluating the inflammatory and immune state of patients with CKD. Therefore, it is assumed that AISI and SII can perform better than more limited indices for predicting the risk of death and transfer to HD. Thus, considering the behavior of leukocytes in the inflammatory and immune process, an increase in the numerator (neutrophils, platelets, and monocytes) and decrease in the denominator (lymphocytes) of AISI and SII may indicate that these indices are capable of reflecting the inflammatory process in patients with CKD in PD and, consequently, can be used to predict mortality and transfer to HD. More studies need to be performed to validate these results.

Although NLR, PLR, and MLR indices are the most investigated in CKD and have already been associated with mortality by all causes, including cardiovascular causes, in several studies, we found no significant association. To date, no study has been conducted in Brazil to investigate the association of hematological indexes with mortality in PD patients, which makes it impossible to compare results. Most studies found in the literature that found a positive association of NLR, PLR, and MLR hematological indices with mortality in CKD are Chinese, and their results have no external validation for other populations. In addition, our study had a small sample size, the age of our population was higher, and follow-up time was shorter compared to the Chinese studies^5,6,8,22,28,29,31,32,33^.

Despite the importance of CKD etiology and its effect on the investigated outcomes, our study identified that the number of peritonitis episodes was a significant variable in all evaluated Cox regression models. The number of peritonitis episodes had HR = 2.34 (95%CI: 1.127; 4.842) in the model that includes AISI, and HR = 1.80 (95%CI: 1.015; 3,182) in the SII adjusted model. This corroborates the prior knowledge that researchers have about the influence of this variable on outcomes. Peritonitis is known to be the main cause of PD failure. LAN et al.^34^ suggested a standardized definition of technical failure, including transfer to HD and death. In a study of 808 patients with an average age of 63.9 years and average PD of 1.89 years, 162 patients were transferred to HD and 74 died, and the main cause of transfer to HD (36%) was peritonitis infection. The general mortality was 5.1/100 patient-years. CVD-related mortality was 1.7/100 patient-years and the mortality related to infection and peritonitis was 0.6 and 0.2/100 patients-years^35^.

The causes of death were not known for some patients in our study. Further studies and evidence are necessary to evaluate other causes of HD transfer and mortality.

The AISI adjusted model included creatinine (HR = 1.38; p = 0.01). Creatinine is used in the calculation of GFR, i.e., the higher its value the greater the impairment of renal function. In the study by Ercan et al.^23^ in which AISI proved to be an important predictor of survival in patients with CKD stages 3-5 hospitalized by COVID-19, the average level of creatinine was 3.59, but this variable was not used in the Cox analysis to verify its effect on survival. Serum creatinine levels in pediatric patients with CKD in pre-dialysis were high in all groups in the Brazilian study by Silva et al.^26^ which included stage 1-2 patients with CKD caused by congenital kidney and urinary tract anomalies (1.15 ± 0.37), stage 3-4 patients with CKD caused by glomerulopathies (3.67 ± 1.71) or congenital anomalies of the kidney and urinary tract (3.02 ± 1.18), and stage 3-4 patients with CKD caused by other etiologies (2.59 ± 0.95). In most studies, creatinine was not analyzed by COX regression models, appearing only within the GFR calculation.

Correlation analyses were performed to compare hematological indices with other classic inflammatory markers evaluated in the study (ferritin, global leukocytes, IL-6, and IL-10). AISI and SIRI showed high and positive correlations, and SII, low and positive correlations with global leukocyte count. AISI, SII, and SIRI are the indices that have the most leukocyte subtypes in their numerator (neutrophils, platelets, and monocytes). Multiple blood cell populations participate in systemic inflammation as mentioned earlier and, therefore, the inclusion of these cells in the calculation of an index better reflects inflammatory state and improves their predictive ability^36^. Proportions between lymphocyte, neutrophil, platelet, and monocyte counts are more strongly associated with chronic inflammation conditions compared to individual cell populations^37^. The PLR index showed a low and negative correlation with the global leukocyte count. PLR is the only index that has a single leukocyte subtype in its formula (lymphocytes), which may explain the negative correlation with global leukocyte count. The indices that have positive correlations with this variable have 2 or 3 leukocyte subtypes in their formulas, which may lead to a behavior similar to that of global leukocyte.

Our study has some limitations. First, the small sample size may affect the estimates obtained, but it represents the population of one of the largest nephrology centers with PD patients in Brazil. Second, as with all observational studies, this study can only reveal associations and not establish causality, and many of our conclusions generate hypotheses. Third, the follow-up time of our study was shorter compared to the studies mentioned in this study, which may have influenced the associations studied. Despite these limitations, this is a pioneering study conducted in Brazil that investigates the association between hematological indices and both all-cause mortality and transfer to HD, contributing to important evidence regarding the use of practical and more affordable tools for risk assessment.

## Conclusion

AISI and SII indices were associated with all-cause mortality and transfer to HD in patients undergoing PD. AISI >149.61 and SII >722.80 were independently associated with the risk of death or transfer to hemodialysis in patients in PD, and in both AISI and SII indices, the number of cases of peritonitis influenced the occurrence of the outcomes.

Hematological indices have been well investigated in the prognosis of many diseases and mortality, however further studies are needed to clarify how these indices can be effectively applied in clinical practice and in which contexts. In addition, the absence of established reference values limits the proper interpretation of their potential clinical value. Therefore, more studies are essential to define these reference values in the general population, maximizing the potential benefits of these accessible and cost-effective prognostic tools.

## Data Availability

The dataset supporting the results of this study is not publicly available.

## Supplementary Material

The following online material is available for this article:

Table S1 – Hypothesis testing for proportional hazards in the model adjusted for the AISI index.

Table S2 – Hypothesis testing for proportional hazards in the model adjusted for the SII index.

Figure S1 – Schoenfeld residual plots for the AISI index model.

Figure S2 – Martingale and deviance residual graphs for the AISI index model.

Figure S3 – Schoenfeld residual plots for the SII index model.

Figure S4 – Martingale and Deviance residual graphs for the SII index model.
