# Supplementary material for: Hematological indices derived from complete blood count and unfavorable outcomes in patients under-going peritoneal dialysis
Source: J Bras Nefrol. 2025 Sep 12;47(4):e20250017. doi: 10.1590/2175-8239-JBN-2025-0017en (PMC12435867; doi:10.1590/2175-8239-JBN-2025-0017en)
Supplement: Supplementary file 2 [file 2175-8239-jbn-47-4-e20250017-suppl1.pdf]

Braz. J. Nephrol.
https://doi.org/10.1590/2175-8239-JBN-2025-0017en

Supplementary Material to "Haematological indices derived from complete blood count and unfavorable outcomes in patients undergoing peritoneal dialysis"

Table S1 - Hypothesis testing for proportional hazards in the model adjusted for the AISI index.

Variable	χ^2	p value
Total time in PD	1.07	0.30
Sex	0.01	0.94
Age	3.41	0.07
HD before PD	0.17	0.68
Number of peritonitis	1.54	0.21
Creatinina	2.35	0.13
AISI	0.03	0.85
Global	4.95	0.67

Abbreviations - PD: peritoneal dialysis; HD: hemodialysis: AISI: Aggregated Index of Systemic Inflammation.
