# Supplementary material for: Hematological indices derived from complete blood count and unfavorable outcomes in patients under-going peritoneal dialysis
Source: J Bras Nefrol. 2025 Sep 12;47(4):e20250017. doi: 10.1590/2175-8239-JBN-2025-0017en (PMC12435867; doi:10.1590/2175-8239-JBN-2025-0017en)
Supplement: Supplementary file 4 [file 2175-8239-jbn-47-4-e20250017-suppl2.pdf]

Braz. J. Nephrol.
https://doi.org/10.1590/2175-8239-JBN-2025-0017en

Supplementary Material to "Haematological indices derived from complete blood count and unfavorable outcomes in patients undergoing peritoneal dialysis"

Table S2 - Hypothesis testing for proportional hazards in the model adjusted for the SII index.

Variable	χ^2	p value
Total time in PD	2.18	0.14
Sex	0.01	0.93
Age	2.11	0.15
HD before PD	0.05	0.82
Number of peritonitis	2.27	0.13
SII	2.91	0.08
Global	6.39	0.38

Abbreviations - PD: peritoneal dialysis; HD: hemodialysis: SII: Systemic Inflammatory Index.
