# Supplementary material for: Hematological indices derived from complete blood count and unfavorable outcomes in patients under-going peritoneal dialysis
Source: J Bras Nefrol. 2025 Sep 12;47(4):e20250017. doi: 10.1590/2175-8239-JBN-2025-0017en (PMC12435867; doi:10.1590/2175-8239-JBN-2025-0017en)
Supplement: Supplementary file 6 [file 2175-8239-jbn-47-4-e20250017-suppl6.pdf]

Braz. J. Nephrol.
https://doi.org/10.1590/2175-8239-JBN-2025-0017en

Supplementary Material to "Haematological indices derived from complete blood count and unfavorable outcomes in patients undergoing peritoneal dialysis"

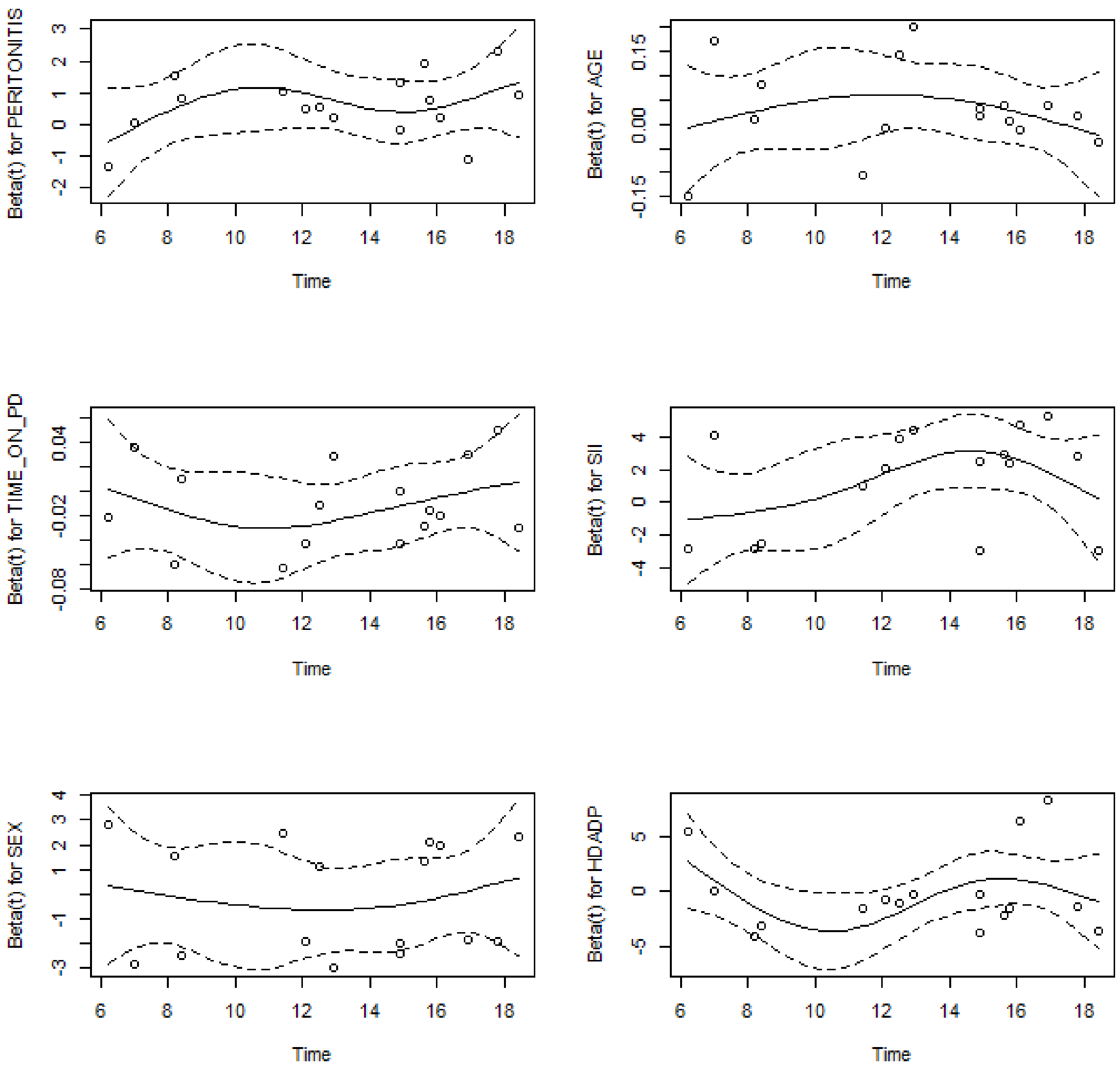

Figure S3 - Schoenfeld residual plots for the SII index model.
