# Supplementary material for: Hematological indices derived from complete blood count and unfavorable outcomes in patients under-going peritoneal dialysis
Source: J Bras Nefrol. 2025 Sep 12;47(4):e20250017. doi: 10.1590/2175-8239-JBN-2025-0017en (PMC12435867; doi:10.1590/2175-8239-JBN-2025-0017en)
Supplement: Supplementary file 8 [file 2175-8239-jbn-47-4-e20250017-suppl7.pdf]

Braz. J. Nephrol.
https://doi.org/10.1590/2175-8239-JBN-2025-0017pt

Material Suplementar para "Índices hematológicos derivados de hemograma completo e desfechos desfavoráveis em pacientes submetidos à diálise peritoneal"

Tabela S1 - Teste de hipóteses para riscos proporcionais no modelo ajustado para o índice AISI.

Variável	χ^2	p valor
Tempo total em DP	1,07	0,30
Sexo	0,01	0,94
Idade	3,41	0,07
HD antes DP	0,17	0,68
Número de peritonites	1,54	0,21
Creatinina	2,35	0,13
AISI	0,03	0,85
Global	4,95	0,67

Abreviações - DP: diálise peritoneal; HD: hemodiálise: AISI: Índice Agregado de Inflamação Sistêmica.
