# Supplementary material for: Hematological indices derived from complete blood count and unfavorable outcomes in patients under-going peritoneal dialysis
Source: J Bras Nefrol. 2025 Sep 12;47(4):e20250017. doi: 10.1590/2175-8239-JBN-2025-0017en (PMC12435867; doi:10.1590/2175-8239-JBN-2025-0017en)
Supplement: Supplementary file 10 [file 2175-8239-jbn-47-4-e20250017-suppl8.pdf]

Braz. J. Nephrol.
https://doi.org/10.1590/2175-8239-JBN-2025-0017pt

Material Suplementar para "Índices hematológicos derivados de hemograma completo e desfechos desfavoráveis em pacientes submetidos à diálise peritoneal"

Tabela S2 - Teste de hipóteses para riscos proporcionais no modelo ajustado para o índice SII.

Variável	χ^2	p valor
Tempo total em DP	2,18	0,14
Sexo	0,01	0,93
Idade	2,11	0,15
HD antes DP	0,05	0,82
Número de peritonites	2,27	0,13
SII	2,91	0,08
Global	6,39	0,38

Abreviações - DP: diálise peritoneal; HD: hemodiálise: SII: Índice Inflamatório Sistêmico.
