# Supplementary material for: Hematological indices derived from complete blood count and unfavorable outcomes in patients under-going peritoneal dialysis
Source: J Bras Nefrol. 2025 Sep 12;47(4):e20250017. doi: 10.1590/2175-8239-JBN-2025-0017en (PMC12435867; doi:10.1590/2175-8239-JBN-2025-0017en)
Supplement: Supplementary file 11 [file 2175-8239-jbn-47-4-e20250017-suppl11.pdf]

Braz. J. Nephrol.

https://doi.org/10.1590/2175-8239-JBN-2025-0017pt

Material Suplementar para "Índices hematológicos derivados de hemograma completo e desfechos desfavoráveis em pacientes submetidos à diálise peritoneal"

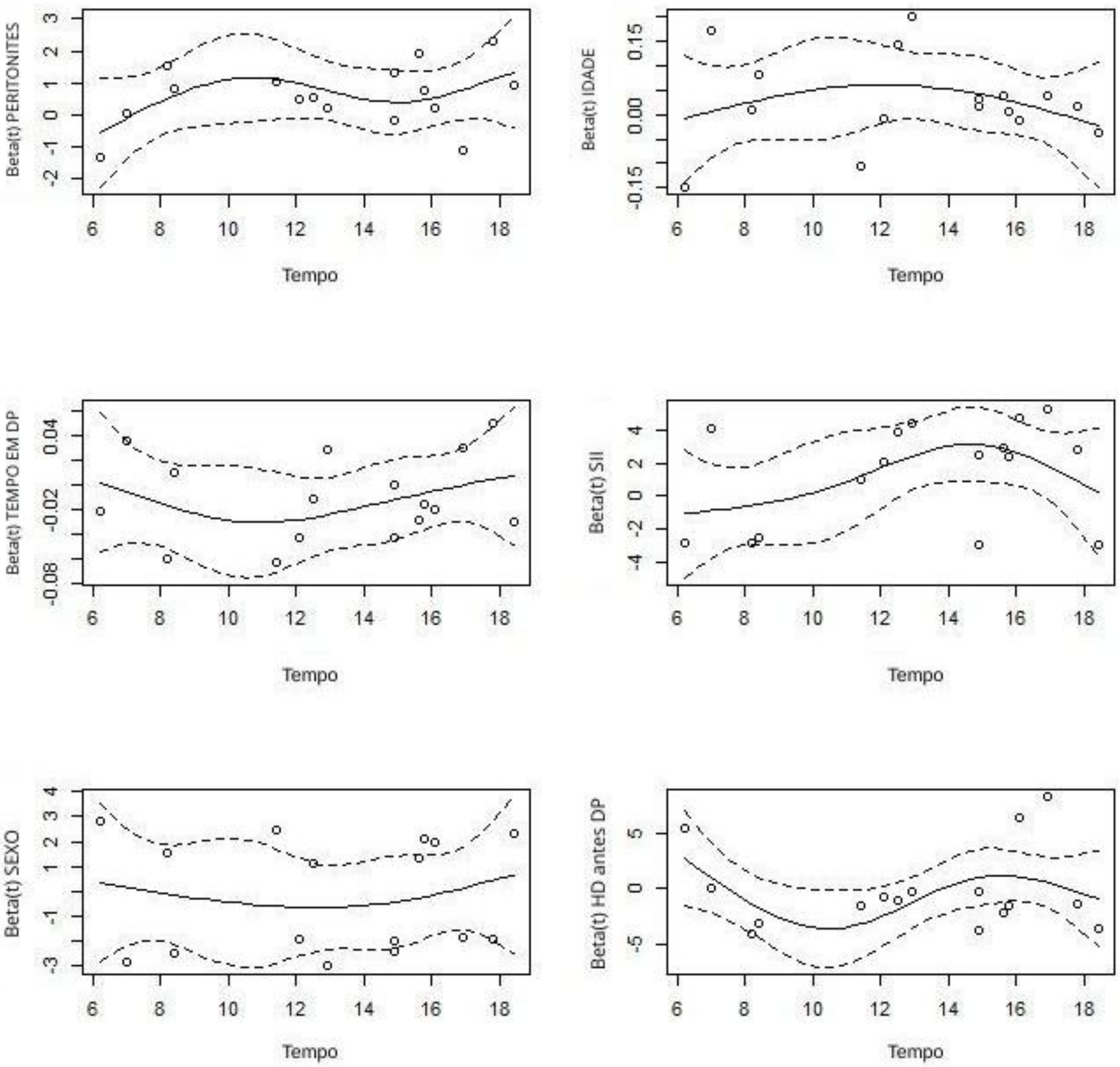

Figura S3 - Gráficos de resíduos de Schoenfeld para o modelo com o índice SII.
