# Supplementary material for: Hematological indices derived from complete blood count and unfavorable outcomes in patients under-going peritoneal dialysis
Source: J Bras Nefrol. 2025 Sep 12;47(4):e20250017. doi: 10.1590/2175-8239-JBN-2025-0017en (PMC12435867; doi:10.1590/2175-8239-JBN-2025-0017en)
Supplement: Translation

# Translation: Índices hematológicos derivados de hemograma completo e desfechos desfavoráveis em pacientes submetidos à diálise peritoneal

**DOI:** 10.1590/2175-8239-JBN-2025-0017pt

**Authors:** Taluane Vívian Gomes Alves, Luciane Teixeira Passos Giarola, Wander Valadares de Oliveira, Danyelle Romana Alves Rios

**Affiliations:** 1 Universidade Federal de São João del-Rei, Divinópolis, MG, Brasil.; 2 Universidade Federal de São João del-Rei, Departamento de Matemática e Estatística, São João del Rei, MG, Brasil.; 3 Universidade do Estado de Minas Gerais, Passos, MG, Brasil.

**Keywords:** Biomarcadores, Diálise Peritoneal, Mortalidade, Modelos de Cox

## Abstract

**Introdução:**

Compreender os processos inflamatórios associados ao risco de mortalidade em pacientes em diálise peritoneal (DP) pode ajudar a orientar a tomada de decisões clínicas e a estratificação de risco e mortalidade nessa população.

**Objetivo:**

Avaliar a associação entre índices hematológicos derivados de hemograma completo e desfechos desfavoráveis em pacientes sob DP.

**Métodos:**

Coorte prospectiva com 43 pacientes em DP acompanhados por 18 meses. Dados do hemograma completo foram coletados dos prontuários médicos e índices hematológicos foram calculados para todos os participantes nas quatro etapas do acompanhamento. Associações entre esses índices e marcadores inflamatórios clássicos foram investigadas por meio de análises de correlação. A sobrevida dos pacientes foi estimada pelo método de Kaplan Meier (K-M) após divisão dos pacientes em dois grupos, com base na mediana como ponto de corte para cada índice hematológico. Utilizou-se o modelo de Cox com estrutura de risco competitivo para avaliar a influência dos índices na sobrevida.

**Resultados:**

Os índices AISI e SIRI apresentaram correlação positiva significativa com a contagem total de leucócitos (r = 0,74 e r = 0,71, respectivamente, p < 0,001). Apenas AISI e SII apresentaram estimativas significativas de K-M, indicando maior sobrevida para AISI ≤149,61 e SII ≤722,80. No modelo de regressão de Cox, os pacientes com AISI superior a 149,6 e SII acima de 722,80 apresentaram risco 9,38 e 4,0 vezes maior, respectivamente, de óbito ou transferência para HD em comparação aos demais pacientes.

**Conclusão:**

AISI e SII foram independentemente associados ao risco de desfechos desfavoráveis em pacientes em DP.

## Introdução

A doença renal crônica (DRC) é um importante problema de saúde pública devido à sua alta prevalência, impacto econômico e elevada morbimortalidade^1^. Embora os tratamentos para a DRC tenham promovido uma melhora da condição ao longo do tempo, a inflamação crônica e o estresse oxidativo nos pacientes aceleram o processo de morbidade e mortalidade^2^.

A diálise peritoneal (DP) é uma das opções de terapia renal substitutiva (TRS) na qual o peritônio, a membrana que reveste os principais órgãos do abdômen, é utilizado para a filtração do sangue. O funcionamento adequado da DP depende da integridade estrutural e funcional da membrana peritoneal. A peritonite (inflamação do peritônio) relacionada à DP permanece a principal complicação e desafio para o sucesso do tratamento a longo prazo e a principal causa de falha da DP e transferência para hemodiálise (HD)^3,4^.

No monitoramento de rotina de pacientes em DP, são necessárias medidas mais acessíveis, convenientes e eficazes para a estratificação do risco de desfechos desfavoráveis^5^. Assim, novos marcadores têm sido caracterizados como potenciais fatores prognósticos para mortalidade por doença cardiovascular (DCV) e mortalidade por todas as causas em pacientes submetidos à DP e HD, bem como para outras condições clínicas^5-8^. Exemplos desses marcadores são os índices hematológicos derivados do hemograma completo, que podem ser obtidos por meio de cálculos simples com base na contagem de neutrófilos, linfócitos, plaquetas e monócitos presentes no sangue^9^. Os índices hematológicos incluem: relação neutrófilos/linfócitos (NLR), relação plaquetas/linfócitos (PLR), relação neutrófilos/linfócitos derivada (dNLR), razão monócitos/linfócitos (MLR), índice agregado de inflamação sistêmica (AISI), índice de inflamação imunológica sistêmica (SII) e índice de resposta inflamatória sistêmica (SIRI)^9,10^.

A inflamação é uma característica comum da DRC, frequentemente associada a níveis elevados de marcadores inflamatórios, incluindo citocinas pró-inflamatórias (IL-6, IL-1 e fator de necrose tumoral alfa [TNF-α]) e citocinas anti-inflamatórias, como IL-10. Em um estudo que avaliou o potencial de diversos biomarcadores inflamatórios no diagnóstico e estadiamento da DRC, os níveis circulantes de IL-6 e TNFR2 estiveram significativamente elevados em todos os estágios da doença^11^. No entanto, esses marcadores não são avaliados rotineiramente devido aos elevados custos envolvidos^5^.

A ferritina sérica, também considerada um marcador inflamatório, pode estar aumentada em condições inflamatórias, como a DRC, independentemente das reservas de ferro, sendo positivamente correlacionada com a gravidade da inflamação^12^. A contagem total de leucócitos, outro marcador inflamatório amplamente utilizado, é empregada em diferentes contextos clínicos^13,14^. Como todas as proporções desses índices são derivadas da contagem total de leucócitos, existe uma relação matemática entre eles. Em um estudo que avaliou índices hematológicos (NLR, MLR, PLR, dNLR, AISI, SIRI e SII) na predição de mortalidade em 1.792 pacientes idosos e não idosos com resultado positivo para COVID-19, os níveis de ferritina e leucócitos foram significativamente mais elevados nos pacientes que foram à óbito em comparação com os sobreviventes^15^.

Compreender os processos inflamatórios associados ao risco de mortalidade em pacientes submetidos à DP é fundamental para o controle da inflamação sistêmica^16^. Para orientar a tomada de decisões clínicas com o objetivo de possibilitar um melhor prognóstico, são necessários recursos terapêuticos e diagnósticos mais acessíveis, capazes de identificar possíveis complicações da doença e o risco de mortalidade^5^. Assim, o presente estudo tem como objetivo avaliar a associação de índices hematológicos derivados do hemograma completo com desfechos desfavoráveis (por mortalidade por todas as causas e transferência para HD) em pacientes submetidos à DP.

## Método

### Desenho e População do Estudo

Este é um estudo prospetivo de coorte com pacientes em DP em um centro de Nefrologia de um município do estado de Minas Gerais. Trata-se de um centro de referência regional para o tratamento de doenças renais e que figura entre os maiores centros de Nefrologia do país. Os participantes foram acompanhados de agosto de 2011 a fevereiro de 2013.

Os pacientes elegíveis foram convidados a participar do estudo após consulta ambulatorial de rotina, realizada uma vez por mês. De um total de 74 pacientes em DP, 43 foram considerados elegíveis. Os critérios de inclusão foram: estar em DP há pelo menos 90 dias, apresentar estabilidade clínica (sem episódios de peritonite, hospitalização ou doenças agudas no mês anterior, durante ou no mês seguinte à avaliação) e ter idade igual ou superior a 18 anos. Os critérios de exclusão foram: doenças agudas, doenças autoimunes, neoplasias, soropositividade para HIV, gestação e impossibilidade de assinar o termo de consentimento livre e esclarecido devido a doença psiquiátrica ou transtorno mental.

A avaliação basal, considerada como a primeira fase do estudo (agosto de 2011), incluiu 43 pacientes. A segunda fase ocorreu em fevereiro de 2012, com uma perda devido a óbito. Na terceira fase, ocorrida em agosto de 2012, houve sete perdas (16,3%) — quatro devido a óbito (9,3%) e três por transferência para HD (7%). A quarta fase aconteceu em fevereiro de 2013 e, durante o acompanhamento, houve oito perdas (20,9%) — cinco óbitos (11,6%) e três transferências para HD (7,0%). Além disso, dois pacientes ficaram debilitados (2,3%), finalizando o estudo com 25 pacientes (Figura 1).

**Figura 1:** População em cada fase do estudo.

### Coleta de Dados

A coleta de dados foi realizada durante as consultas de rotina, realizadas a cada seis meses. As informações foram coletadas dos prontuários médicos dos pacientes, incluindo dados sociodemográficos, condições de saúde, exames laboratoriais de rotina, uso de medicamentos, registro de peritonite, complicações ocorridas durante o período da pesquisa, tempo total em DP e outras terapias dialíticas e data do óbito. Informações sobre mortalidade, hora do óbito e tempo decorrido até a transferência para HD foram extraídas dos registros hospitalares disponíveis e, posteriormente, verificadas no Sistema de Informações Nefrodata da Unidade de Nefrologia do Hospital.

Os dados do hemograma completo também foram coletados a partir dos prontuários médicos. Os índices hematológicos foram calculados para todos os participantes nas quatro etapas de acompanhamento, a saber: NLR: relação neutrófilos/linfócitos; dNLR: neutrófilos/leucócitos totais – neutrófilos; PLR: plaquetas/linfócitos; MLR: monócitos/linfócitos; AISI: neutrófilos × monócitos × plaquetas/linfócitos; SII: plaquetas × neutrófilos/linfócitos; e SIRI: neutrófilos × monócitos/linfócitos.

Também foram coletadas as seguintes variáveis: sexo, idade, doença primária, tempo total em DP, tempo de participação no estudo, número de episódios de peritonite, HD anterior à DP, tempo em terapia dialítica (HD ou DP), transferência para HD, tempo até o óbito (desde o início do estudo até o óbito), índice de massa corporal (IMC), presença de diabetes mellitus e hipertensão, pressão arterial sistólica e diastólica, glicemia de jejum, creatinina, ferro sérico, capacidade total de ligação do ferro, índice de saturação da transferrina, ferritina, colesterol total e frações, triglicerídeos, alanina aminotransferase, fosfatase alcalina, proteína total, albumina, globulina, cálcio, fósforo, potássio, paratormônio (PTH).

O estudo foi aprovado pelos Comitês de Ética em Pesquisa da Universidade Federal de São João Del-Rei e do Hospital São João de Deus-CAAE-19284613.5.0000.5545.

### Análise Estatística

Os dados descritivos foram apresentados como número e frequência (%) para dados categóricos, média e desvio padrão para dados contínuos com distribuição normal e mediana e intervalos interquartis para dados assimétricos. O teste de Shapiro-Wilk foi utilizado para testar a normalidade dos dados. O teste de correlação de Pearson foi empregado para avaliar a associação entre marcadores inflamatórios clássicos (ferritina, leucócitos totais, IL-6 e IL-10) e índices hematológicos.

A sobrevida dos pacientes foi estimada pelo método de Kaplan Meier. Esse método também foi utilizado para estimar a sobrevida dos pacientes quando dicotomizados pela mediana de cada um dos índices hematológicos: menor ou igual à mediana e superior à mediana. A comparação das curvas de sobrevida entre os grupos de cada índice foi realizada por meio do teste log-rank.

Para avaliar a influência dos índices hematológicos na sobrevida, foi utilizado o modelo de riscos proporcionais de Cox com abordagem de riscos competitivos. Dois desfechos foram considerados: óbito e transferência para tratamento com HD. O procedimento analítico seguiu a caracterização análoga ao modelo clássico de regressão de Cox^17^. Dessa forma, foram construídos modelos considerando um dos sete índices hematológicos em cada modelo, além das covariáveis sexo, idade, tempo total em DP, número de episódios de peritonite, HD anterior à DP, IMC, presença de diabetes mellitus, pressão arterial sistólica, pressão arterial diastólica, glicemia de jejum, creatinina e hipertensão arterial.

Uma seleção prévia das variáveis do modelo foi realizada pelo processo “*backward*”, utilizando o Critério de Informação de Akaike (AIC, em inglês), considerando as variáveis que compõem o modelo com o menor valor de AIC. A significância dessas variáveis foi avaliada pelo teste de Wald^18^. Aquelas que não apresentaram significância estatística foram excluídas do modelo, uma a uma, até que se obtivesse um modelo no qual todas as variáveis influenciassem o risco dos desfechos. A partir dessa etapa, as variáveis anteriormente excluídas foram reintegradas, uma a uma, no modelo obtido, e sua significância foi novamente avaliada pelo teste de Wald, bem como pelo teste da razão de verossimilhança, ao comparar os modelos com e sem a variável mencionada. As variáveis reconhecidamente importantes na prática clínica por explicarem óbito ou transferência para HD foram incluídas no modelo final, mesmo que não apresentassem significância estatística. As estimativas dos parâmetros, realizadas pelo método da máxima verossimilhança parcial, referem-se ao efeito comum (média) das variáveis para os dois desfechos.

Gráficos de resíduos de Schoenfeld foram construídos para avaliar a suposição de riscos proporcionais do modelo de Cox, utilizando o coeficiente de correlação de Pearson entre os resíduos e o tempo, sendo realizado um teste de hipótese para essa correlação. São desejáveis valores elevados de probabilidade de significância, uma vez que indicam riscos proporcionais. Resíduos Martingale e Deviance foram utilizados para avaliar a adequação do modelo ajustado.

As análises foram realizadas com um nível de significância de 5% e executadas utilizando o software R (2023), com o auxílio dos pacotes “SURVIVAL” e “MASS”, e o software STATA versão 14.0^19,20^.

## Resultados

As características basais da população do estudo estão apresentadas na Tabela 1. A média de idade dos pacientes foi de 63 anos, 51,2% eram do sexo masculino e a mediana do IMC foi de 24,6. Dez participantes (23,3%) já haviam sido submetidos à terapia de HD antes de mudar para DP. A pressão arterial sistólica média foi de 142,0 e a mediana da pressão arterial diastólica foi de 80,0. Entre as etiologias conhecidas, a principal causa de DRC foi diabetes mellitus (30,3%).

**Tabela 1:** Características sociodemográficas, clínicas e laboratoriais da população do estudo

Variáveis	Total	Valores de referência
Idade (Anos)	63,0 ± 15,3	
Sexo		
Masculino [n (%)]	22 (51,2%)	
IMC (kg/m^2^)	24,6 (21,3; 26,3)	
Principais causas de DRC [n (%)]		
Diabetes	13 (30,3%)	
Hipertensão arterial sistêmica	7 (16,3%)	
GNC	2 (4,6%)	
DRP, CAKUT	3 (7%)	
Etiologias desconhecidas	18 (41,8%)	
Pressão sistólica (mmHg)	142,0 ± 21,4	
Pressão diastólica (mmHg)	80 (80; 80)	
Total de pacientes com peritonite registrados (n) ao longo de 18 meses [n (%)]		
0 episódios de peritonite	28 (65,1%)	
1 episódio de peritonite	12 (57,1%)	
2 episódios de peritonite	1 (4,8)	
3 episódios de peritonite	1 (4,8%)	
4 episódios de peritonite	1 (4,8%)	
Pacientes com peritonite [n (%)]	15 (34,9%)	
Tempo total em DP (meses)	46,4 (31,0; 62,7)	
HD anterior à DP [n (%)]	10 (23,3%)	
Tempo em terapia com HD ou DP (meses)	45,8 ± 20,3	
Tempo de participação no estudo (meses)	16,4 ± 4,1	
Óbitos [n (%)]	10 (23,3%)	
Tempo transcorrido até o óbito (meses)	12,5 ± 4,2	
Transferência para HD [n (%)]	6 (13,9%)	
Em DP por mais de 36 meses [n (%)]	17 (39,5%)	
Glicemia de jejum (mg/dL)	102 (82; 146)	Inferior a 99 mg/dL
Creatinina sérica (mg/dL)	9,47 ± 4,26	M: 1 (0,7 a 1,2 mg/dL) F: 0,8 (0,5 a 1,0 mg/dL)
Ferro sérico (mcg/dL)	64,88 ± 26,3	M: 65 mcg/dL a 175 mcg/dL F: 50 mcg/dL a 170 mcg/dL
Capacidade total de ligação do ferro (mcg/dL)	209 (189; 253)	M: 69 mcg/dL a 240 mcg/dL F: 70 mcg/dL a 310 mcg/dL
Índice de saturação da transferrina (%)	27,6 ± 13,3	20,0 a 55,0%
Ferritina (ng/mL)	169 (100; 482)	M: 23 a 336 ng/mL F: 12 a 306 NG/mL
Colesterol total (mg/dL)	188,9 ± 66,0	< 190 mg/dL
HDL-C (mg/dL)	40 (34; 48)	M: > 40 mg/dL F: > 50 mg/dL
LDL-C (mg/dL)	114,4 ± 53,9	<130 mg/dL
Triglicerídeos (mg/dL)	150 (110; 208)	<150 mg/dL
Alanina aminotransferase (U/L)	19 (17; 25)	M: < 45 U/L F: < 34 U/L
Fosfatase alcalina (U/L)	93 (79; 117)	40 U/L a 150 U/L
Proteína total (g/dL)	6,1 ± 0,6	6,0 a 8,0 g/dL
Albumina (g/dL)	3,2 ± 0,4	3,5 g/dL a 5,2 g/dL
Globulina (g/dL)	2,9 ± 0,4	1,5 a 3,5 g/dL
Cálcio (mg/dL)	9,3 ± 0,7	8,8 a 10,4 mg/dL
Fósforo (mg/dL)	5 (4,1; 6,4)	M: 2,4 a 4,6 mg/dL F: 2,3 a 4,3 mg/dL
Potássio (mmol/L)	4,5 ± 0,8	3,5 a 5,0 mmol/L
Paratormônio (pg/mL)	184 (68; 375)	12,0 pg/ml a 88,0 pg/ml
Hemoglobina (g/dL)	11,7 ± 1,9	M: 14,9 g/dL (13,0–16,9) F: 13,2 g/dL (11,5–14,9)
Contagem total de leucócitos (x 10^9^/L)	6,4 ± 2,5	4,0 a 10,0 x 10^9^/L
Neutrófilos (x 10^9^/L)	4,4 ± 1,7	2,0 a 7,0 x 10^9^/L
Bandas (x 10^9^/L)	0,2 (0;0)	0 a 1,2 x 10^9^/L
Monócitos (x 10^9^/L)	0,2 (0,2; 0,3)	0,2 a 1,2 x 10^9^/L
Basófilos (x 10^9^/L)	0,6 (0;0)	0 a 0,22 x 10^9^/L
Linfócitos (x 10^9^/L)	1,4 (1,2; 1,9)	1,0 a 3,0 x 10^9^/L
Plaquetas (x 10^9^/L)	219,9 ± 81,7	150 a 450 x 10^9^/L

Abreviações – IMC: índice de massa corporal; DRC: doença renal crônica; GNC: glomerulonefrite crônica; DRP: doença renal policística; CAKUT: anomalias congênitas do rim e do trato urinário; DP: diálise peritoneal; HD: hemodiálise; HDL-C: colesterol de lipoproteína de alta densidade; LDL-C: colesterol de lipoproteína de baixa densidade; F: feminino; M: masculino.

Notas – Os resultados são apresentados como média e desvio padrão para dados com distribuição normal, de acordo com o teste de Shapiro-Wilk, e mediana (quartis) para dados com distribuição assimétrica. As variáveis categóricas são apresentadas como proporções: n (%).

A duração média da DP foi de 53,5 meses, com 50% dos pacientes apresentando uma duração de até 46,4 meses. Foram registrados 21 episódios de peritonite, com 15 (34,9%) pacientes apresentando peritonite durante o período de monitoramento. A duração média da participação dos pacientes no estudo foi de 16,4 meses. Ao final do período de acompanhamento, houve 10 (23,3%) óbitos e 6 (13,9%) pacientes foram transferidos para HD.

A Tabela 2 apresenta os resultados obtidos para os coeficientes de correlação de Pearson (r) entre os marcadores inflamatórios clássicos (ferritina, contagem total de leucócitos, IL-6 e IL-10) e os índices hematológicos na avaliação basal. Os índices AISI e SIRI apresentaram uma correlação elevada, positiva e estatisticamente significativa com a contagem total de leucócitos (r = 0,74 e r = 0,71, respectivamente). O índice PLR apresentou uma correlação baixa, negativa e significativa com a contagem total de leucócitos (r = -0,32). O índice SII apresentou uma correlação baixa, positiva e significativa com a contagem total de leucócitos (r = 0,45). Os demais índices não apresentaram correlações estatisticamente significativas. A escala de correlação utilizada refere-se aos valores da matriz de correlação, sendo classificados como muito baixos para valores iguais ou inferiores a 0,25, baixos para valores de 0,26 a 0,49, moderados entre 0,50 e 0,69, altos entre 0,70 e 0,89 e muito altos entre 0,90 e 1,00^21^.

**Tabela 2:** Análise de correlação entre os marcadores ferritina, contagem total de leucócitos, il-6 e il-10 e os índices hematológicos no início do estudo

Marcadores	Ferritina	Contagem total de leucócitos	IL-6	IL-10
NLR	–0,14	0,22	–0,14	–0,09
dNLR	–0,17	0,19	–0,10	0,01
PLR	–0,11	–0,32*	–0,07	0,10
MLR	–0,15	0,16	–0,18	0,07
AISI	–0,19	0,74*	–0,11	–0,07
SII	–0,17	0,45*	–0,15	–0,03
SIRI	–0,20	0,71*	–0,17	–0,10

Abreviações – NLR: relação neutrófilos/linfócitos; dNLR: relação neutrófilos/linfócitos derivada; PLR: relação plaquetas/linfócitos; MLR: razão monócitos/linfócitos; AISI: Índice Agregado de Inflamação Sistêmica; SII: Índice Inflamatório Sistêmico; SIRI: Índice de Resposta Inflamatória Sistêmica; IL-6: interleucina 6; IL-10: interleucina 10.

Nota – *p < 0,001.

A Figura 2 apresenta as estimativas de sobrevida dos 43 pacientes, obtidas pelo estimador de Kaplan Meier. Nessa figura, é possível observar que as estimativas de sobrevida apresentaram valores acima de 0,6. Esse resultado pode ser explicado pelo curto período de acompanhamento dos pacientes (cerca de 18 meses).

**Figura 2:** Curva de estimativa de sobrevida de Kaplan-Meier.

A Figura 3 mostra as estimativas de sobrevida a partir do estimador de Kaplan Meier para cada um dos grupos obtidos segundo os valores medianos basais de cada um dos índices. As medianas para cada índice foram: NLR = 3,17; dNLR = 3,76; PLR = 168,48; MLR = 0,15; AISI = 149,61; SII = 722,80; e SIRI = 0,70. Para os índices AISI (p = 0,01) e SII (p = 0,02), a sobrevida foi maior nos pacientes abaixo do valor mediano, sendo estatisticamente significativa. NLR, dNLR, PLR, MLR e SIRI não foram estatisticamente significativos (p = 0,58; p = 0,62; p = 0,19; p = 0,33; e p = 0,15, respectivamente).

**Figura 3:** Curvas de Kaplan-Meier das estimativas de sobrevida para todos os índices categorizados de acordo com os valores medianos.

Considerando o modelo de Cox para riscos competitivos, foi possível obter o ajuste para os índices AISI e SII por meio de dois modelos. Os demais índices não apresentaram significância estatística para o risco de ocorrência dos desfechos. Os modelos ajustados incluíram as variáveis de interesse, independentemente de sua significância.

As estimativas do modelo final ajustado para o índice AISI são apresentadas na Tabela 3, juntamente com a respectiva razão de risco (HR) e os resultados obtidos para o teste de Wald. A duração total da DP (p = 0,03), a idade (p = 0,01), o nível de creatinina (p < 0,01) e o número de episódios de peritonite (p = 0,02) foram estatisticamente significativos. O tempo total em DP e a idade apresentaram uma razão de risco próxima de 1 (HR = 0,97 e HR = 1,09, respectivamente). A cada mês adicional em DP, o risco de óbito ou transferência para hemodiálise foi reduzido em 3%. A cada ano de vida, o risco de óbito ou transferência para HD aumentou em 9%. Cada aumento de 1 unidade na creatinina aumentou o risco de óbito ou transferência para HD em 38% (HR = 1,38; p = 0,01). A cada episódio de peritonite diagnosticado, o risco de óbito ou transferência para HD aumentou em 2,34 vezes. Os pacientes com AISI superior a 149,6 apresentaram risco 9,38 vezes maior de óbito ou transferência para HD em comparação aos pacientes com AISI de até 149,6. As variáveis sexo e HD anterior à DP não foram estatisticamente significativas.

**Tabela 3:** Estimativas obtidas para os parâmetros do modelo ajustado, considerando os riscos competitivos de óbito e transferência para hemodiálise e o índice aisi

Parâmetro	Estimativa	Desvio	Estatística de Wald	Valor de p	HR	IC 95% (HR)
Tempo total em DP	–0,03	0,01	–2,20	0,03*	0,97	(0,953; 0,997)
Sexo	0,01	0,54	0,01	0,99	1,01	(0,352; 2,880)
Idade	0,08	0,03	2,56	0,01*	1,09	(1,020; 1,161)
HD anterior à DP	–1,09	0,99	–1,11	0,27	0,34	(0,049; 2,324)
Número de episódios de peritonite	0,85	0,37	2,28	0,02*	2,34	(1,127; 4,842)
Creatinina	0,32	0,11	3,01	< 0,01*	1,38	(1,119; 1,702)
AISI	2,24	0,78	2,86	< 0,01*	9,38	(2,025; 43,420)

Abreviações – DP: diálise peritoneal; HD: hemodiálise; AISI: Índice Agregado de Inflamação Sistêmica.

Nota – *Significativo a 5%.

Para a avaliação da suposição de riscos proporcionais, a Figura S1 apresenta os gráficos de resíduos de Schoenfeld e a Tabela S1 exibe os resultados do teste de hipótese para o coeficiente de correlação de Pearson entre os resíduos de Schoenfeld e o tempo para cada variável do modelo. Não foram observadas violações do risco proporcional (valores p superiores a 0,05). Embora a variável idade tenha apresentado um valor mais baixo (0,07), não houve evidência de violação da proporcionalidade dos riscos em todas as covariáveis, considerando o valor global (0,67) para esta avaliação.

Os gráficos de resíduos Martingale e Deviance, construídos para avaliar a qualidade geral do modelo ajustado, indicam a adequação do modelo, uma vez que os resíduos estão distribuídos aleatoriamente em torno de zero (Figura S2), com os resíduos Martingale inferiores a 1 e os resíduos Deviance entre (-2, 2).

As estimativas do modelo final ajustado para SII, suas respectivas razões de risco e os resultados obtidos pelo teste de Wald são apresentados na Tabela 4. Para cada episódio de peritonite diagnosticado, o risco de óbito ou transferência para HD aumentou em 80% (p = 0,04). Os pacientes com valores de SII acima de 722,8 apresentaram um risco de óbito ou transferência para HD quatro vezes maior do que os demais pacientes (p = 0,03). O tempo total em DP, sexo, idade e HD anterior à DP não apresentaram valores estatisticamente significativos.

**Tabela 4:** Estimativas obtidas para os parâmetros do modelo ajustado, considerando os riscos competitivos de óbito e transferência para hemodialise e o índice sii

Parâmetro	Estimativa	Desvio Erro padrão	Estatística de wald	Valor de p	HR	IC 95% (HR)
Tempo total em DP	–0,01	0,01	–1,18	0,24	0,99	(0,971; 1,007)
Sexo	–0,17	0,53	–0,32	0,75	0,84	(0,297; 2,394)
Idade	0,03	0,02	1,26	0,21	1,03	(0,985; 1,072)
HD anterior à DP	–0,25	0,72	–0,34	0,73	0,78	(0,190; 3,217)
Número de episódios de peritonite	0,59	0,29	2,01	0,04*	1,80	(1,015; 3,182)
SII	1,39	0,65	2,14	0,03*	4,01	(1,127; 14,276)

Abreviações – DP: diálise peritoneal; HD: hemodiálise; SII: Índice Inflamatório Sistêmico.

A Tabela S2 apresenta os resultados do teste de hipótese para o coeficiente de correlação de Pearson entre os resíduos de Schoenfeld e o tempo, e a Figura S3 mostra os gráficos desses resíduos ao longo do tempo. O índice SII, embora tenha apresentado um valor baixo (0,08), pode indicar riscos proporcionais, uma vez que esse valor de p é superior a 5% e o gráfico corrobora tal afirmação. As demais variáveis também não apresentaram evidências de violação da suposição de riscos proporcionais. Além disso, a avaliação global da proporcionalidade confirmou que essa suposição não foi violada (valor p de 0,38).

Os gráficos de resíduos Martingale e Deviance, criados para avaliar a adequação geral do modelo ajustado, indicam conformidade, uma vez que os resíduos estão distribuídos aleatoriamente em torno de zero (Figura S4), com os resíduos Martingale inferiores a 1 e os resíduos Deviance variando entre -3 e 3.

## Discussão

O presente estudo identificou que, em um período médio de acompanhamento de 16,4 meses, foram registrados 10 óbitos (23,3%) e seis (13,9%) transferências para HD, indicando um comprometimento considerável na taxa de sobrevida dos pacientes em DP, considerando o tempo médio de acompanhamento. A taxa de mortalidade foi semelhante à do estudo de Liu et al.^6^, no qual 25,5% dos 939 pacientes em DP foram a óbito em um período mediano de acompanhamento de 27,5 meses. Lu et al.^22^, entretanto, constataram que 43% dos 86 pacientes em DP foram a óbito durante o período do estudo, cuja duração média foi de 25,6 meses.

Também foram obtidas estimativas de sobrevida para cada um dos índices hematológicos. Pacientes em DP com AISI ≤ 149,61 e SII ≤ 722,80 apresentaram maior taxa de sobrevida em comparação com pacientes com valores acima da média (p = 0,01; p = 0,02, respectivamente). Com base nos modelos de Cox, os índices AISI (IC 95% (HR): 2,024; 43,420) e SII (IC 95% (HR): 1,127; 14,276) foram independentemente associados ao aumento do risco de óbito ou transferência para HD. Os amplos intervalos de confiança de ambos os índices podem ser explicados pela pequena dimensão da amostra do estudo.

Até o momento, nenhum estudo havia investigado a associação entre AISI e mortalidade na DRC. Um valor de AISI ≥ 621,1 mostrou-se um preditor de sobrevida em 85 pacientes hospitalizados com COVID-19 e DRC em estágios 3-5, com média de idade de 72,1 anos (HR: 1,001; IC 95%: 1-1,001)^23^. O AISI baseia-se na contagem de linfócitos, neutrófilos, plaquetas e monócitos. Os papéis distintos que essas células desempenham na resposta imune contribuem para sua capacidade de predizer o risco de mortalidade em pacientes com COVID-19 e outras patologias^23^.

Os linfócitos atuam na resposta imune adaptativa, reconhecendo e destruindo antígenos no organismo. A redução dos linfócitos circulantes está associada a infecções recentes, uso de medicamentos específicos e condições de saúde relacionadas ao sistema imunológico. A linfocitopenia leva a um estado inflamatório persistente, pois, além do sistema imunológico, os linfócitos também são responsáveis pela regulação da inflamação, atuando de forma bidirecional com os macrófagos, estimulando-se mutuamente e amplificando a resposta inflamatória^24^.

O aumento de neutrófilos circulantes sugere uma resposta inflamatória aguda ou crônica. Durante a inflamação sistêmica causada por infecções, o aumento dos neutrófilos também estimula a produção de mediadores inflamatórios, como a IL-1A, os quais estimulam a produção de plaquetas por megacariócitos, elevando os níveis de plaquetas circulantes e contribuindo para respostas inflamatórias e imunológicas^25^. Os neutrófilos podem causar inflamação no parênquima renal e inibir a atividade dos linfócitos por meio da supressão da resposta de células T reguladoras e ativação de macrófagos^26^.

Os monócitos são células produzidas na medula óssea, altamente reativas em processos inflamatórios. Ao migrarem para os tecidos, transformam-se em macrófagos, atuando na resposta imunológica e, em resposta a um estímulo, podem intensificar ou reduzir a inflamação^23,26^. Além disso, foi descrito que a presença de frações de monócitos em estado estacionário aumenta de forma consistente durante a inflamação^27^.

Ainda há poucas evidências sobre o papel do SII na DRC, especificamente em pacientes em DP, o que impossibilita a comparação dos resultados encontrados. O SII ≥ 1.063,39 provou ser um fator de risco independente para mortalidade por todas as causas (HR: 1,48; IC 95%: 1,35–1,62), causas cardiovasculares (HR: 1,64; IC 95%: 1,44–1,85) e causas oncológicas (HR: 1,50; IC 95%: 1,09–2,08) em 19.327 pacientes chineses com DRC não submetidos a tratamento dialítico (TFG estimada: <60 ml/min/1,73m^2^), acompanhados por aproximadamente 4,5 anos^28^. Um estudo realizado no Brasil com pacientes com DRC nos estágios 1 a 4, no período pré-diálise, com até 18 anos de idade, avaliou a capacidade das taxas de NLR, dNLR, LMR (relação linfócitos-monócitos), SIRI, AISI e SII na predição da gravidade da doença. Apenas LMR e SIRI apresentaram resultados promissores na avaliação da inflamação; para SII, não houve diferença ao comparar grupos com DRC entre si ou em relação aos controles^26^. O SII já havia sido identificado como um preditor do tratamento da peritonite aguda em um estudo com 138 pacientes chineses com DRC (OR: 0,999; IC 95%: 0,998 –1,000), sugerindo um papel diferente do esperado, atuando como fator protetor no prognóstico de pacientes com peritonite aguda^29^.

Com a progressão da DRC e a deterioração da função renal, o processo inflamatório no parênquima renal se intensifica. Além disso, outras doenças e condições de saúde comumente presentes nesses indivíduos podem agravar o nível de inflamação no organismo^26,30^. Assim, índices hematológicos obtidos por uma combinação mais elevada de células, como AISI (neutrófilos, monócitos, plaquetas e linfócitos) e SII (plaquetas, neutrófilos e linfócitos), podem ser mais úteis na avaliação do estado inflamatório e imunológico de pacientes com DRC. Portanto, presume-se que o AISI e o SII possam ter um desempenho superior ao de índices mais limitados na predição do risco de óbito e transferência para HD. Dessa forma, considerando o comportamento dos leucócitos no processo inflamatório e imunológico, um aumento no numerador (neutrófilos, plaquetas e monócitos) e uma redução no denominador (linfócitos) do AISI e do SII podem indicar que esses índices são capazes de refletir o processo inflamatório em pacientes com DRC em DP e, consequentemente, podem ser empregados para predizer mortalidade e transferência para HD. Estudos adicionais são necessários para validar esses resultados.

Embora os índices NLR, PLR e MLR sejam os mais investigados na DRC e já tenham sido associados à mortalidade por todas as causas, incluindo causas cardiovasculares, em diversos estudos, não encontramos associação significativa. Até o momento, nenhum estudo foi realizado no Brasil para investigar a associação entre índices hematológicos e mortalidade em pacientes em DP, o que impossibilita a comparação de resultados. A maioria dos estudos encontrados na literatura que identificaram uma associação positiva entre os índices hematológicos NLR, PLR e MLR e a mortalidade na DRC são chineses, e seus resultados não possuem validação externa para outras populações. Além disso, nosso estudo contou com uma amostra pequena, os participantes eram mais velhos e o tempo de acompanhamento foi mais curto em comparação aos estudos chineses^5,6,8,22,28,29,31–33^.

Apesar da importância da etiologia da DRC e seu efeito nos desfechos investigados, nosso estudo identificou que o número de episódios de peritonite foi uma variável significativa em todos os modelos de regressão de Cox avaliados. O número de episódios de peritonite apresentou HR = 2,34 (IC 95%: 1,127; 4,842) no modelo que inclui o AISI, e HR = 1,80 (IC 95%: 1,015; 3,182) no modelo ajustado para o SII. Isso corrobora o conhecimento prévio dos pesquisadores quanto à influência dessa variável nos desfechos. A peritonite é reconhecida como a principal causa de falha da DP. LAN et al.^34^ sugeriram uma definição padronizada de falha técnica, incluindo transferência para HD e óbito. Em um estudo envolvendo 808 pacientes com idade média de 63,9 anos e tempo médio em DP de 1,89 anos, 162 pacientes foram transferidos para HD e 74 foram a óbito. A principal causa de transferência para HD (36%) foi infecção por peritonite. A mortalidade geral foi de 5,1/100 pacientes-ano. A mortalidade relacionada a DCV foi de 1,7/100 pacientes-ano e a mortalidade relacionada a infecção e peritonite foi de 0,6 e 0,2/100 pacientes-ano^35^.

As causas do óbito não foram identificadas em alguns pacientes do nosso estudo. Estudos adicionais e evidências são necessários para avaliar outras causas de transferência para HD e de mortalidade.

O modelo ajustado para o AISI incluiu a creatinina (HR = 1,38; p = 0,01). A creatinina é utilizada no cálculo da TFG, ou seja, quanto maior seu valor, maior o comprometimento da função renal. No estudo de Ercan et al.^23^ no qual o AISI se mostrou um importante preditor de sobrevida em pacientes com DRC estágios 3-5 hospitalizados por COVID-19, o nível médio de creatinina foi de 3,59; porém, essa variável não foi incluída na análise de Cox para verificar seu efeito na sobrevida. Os níveis séricos de creatinina em pacientes pediátricos com DRC, em pré-diálise, encontraram-se elevados em todos os grupos no estudo brasileiro de Silva et al.^26^. O estudo incluiu pacientes nos estágios 1-2 com DRC causada por anomalias congênitas do rim e do trato urinário (1,15 ± 0,37), pacientes nos estágios 3-4 com DRC causada por glomerulopatias (3,67 ± 1,71) ou anomalias congênitas do rim e do trato urinário (3,02 ± 1,18), e pacientes nos estágios 3-4 com DRC causada por outras etiologias (2,59 ± 0,95). Na maioria dos estudos, a creatinina não foi analisada pelos modelos de regressão de Cox, aparecendo somente no cálculo da TFG.

Foram realizadas análises de correlação para comparar os índices hematológicos com outros marcadores inflamatórios clássicos avaliados no estudo (ferritina, contagem total de leucócitos, IL-6 e IL-10). Os índices AISI e SIRI apresentaram correlações altas e positivas, e o índice SII apresentou correlações baixas e positivas com a contagem total de leucócitos. AISI, SII e SIRI são os índices que apresentam o maior número de subtipos de leucócitos no numerador (neutrófilos, plaquetas e monócitos). Diversas populações de células sanguíneas participam da inflamação sistêmica, conforme mencionado anteriormente e, portanto, a inclusão dessas células no cálculo de um índice reflete de forma mais acurada o estado inflamatório e melhora sua capacidade preditiva^36^. As proporções entre as contagens de linfócitos, neutrófilos, plaquetas e monócitos estão mais fortemente associadas a condições de inflamação crônica em comparação com a análise individual das populações celulares^37^. O índice PLR demonstrou uma correlação baixa e negativa com a contagem total de leucócitos. O PLR é o único índice que possui um único subtipo de leucócitos em sua fórmula (linfócitos), o que pode explicar a correlação negativa com a contagem total de leucócitos. Os índices que apresentam correlações positivas com essa variável possuem 2 ou 3 subtipos de leucócitos em suas fórmulas, o que pode levar a um comportamento semelhante ao da contagem total de leucócitos.

Nosso estudo apresenta algumas limitações. Primeiro, o pequeno tamanho da amostra pode afetar as estimativas obtidas; no entanto, ela representa a população de um dos maiores centros de nefrologia com pacientes em DP no Brasil. Segundo, como em todos os estudos observacionais, este estudo pode apenas revelar associações e não estabelecer causalidade, sendo que muitas de nossas conclusões geram hipóteses. Em terceiro lugar, o tempo de acompanhamento do nosso estudo foi mais curto em comparação com os estudos mencionados, o que pode ter influenciado as associações analisadas. Apesar dessas limitações, este é um estudo pioneiro realizado no Brasil que investiga a associação entre índices hematológicos e tanto mortalidade por todas as causas quanto transferência para HD, contribuindo com evidências importantes sobre o uso de ferramentas práticas e mais acessíveis para a avaliação de risco.

## Conclusão

Os índices AISI e SII foram associados à mortalidade por todas as causas e à transferência para HD em pacientes submetidos à DP. Valores de AISI >149,61 e SII >722,80 foram independentemente associados ao risco de óbito ou transferência para hemodiálise nesses pacientes e, em ambos os índices, o número de casos de peritonite influenciou a ocorrência dos desfechos.

Os índices hematológicos têm sido amplamente investigados no prognóstico de diversas doenças e na mortalidade; no entanto, estudos adicionais são necessários para esclarecer de que forma esses índices podem ser efetivamente aplicados na prática clínica e em quais contextos. Além disso, a ausência de valores de referência estabelecidos limita a interpretação adequada do seu potencial valor clínico. Portanto, estudos adicionais são essenciais para definir tais valores de referência na população em geral, de modo a maximizar os potenciais benefícios destas ferramentas prognósticas acessíveis e com boa relação custo-benefício.

## Data Availability

O conjunto de dados que fundamenta os resultados deste estudo não está disponível publicamente.

## Material Suplementar

O seguinte material online está disponível para o presente artigo:

Tabela S1 – Teste de hipóteses para riscos proporcionais no modelo ajustado para o índice AISI.

Tabela S2 – Teste de hipóteses para riscos proporcionais no modelo ajustado para o índice SII.

Figura S1 – Gráficos de resíduos de Schoenfeld para o modelo com o índice AISI.

Figura S2 – Gráficos de resíduos Martingale e Deviance para o modelo com o índice AISI.

Figura S3 – Gráficos de resíduos de Schoenfeld para o modelo com o índice SII.

Figura S4 – Gráficos de resíduos Martingale e Deviance para o modelo com o índice SII.
